# Robotic-Assisted Cholecystoduodenal Fistula and Bile Leak Repair

**DOI:** 10.7759/cureus.66583

**Published:** 2024-08-10

**Authors:** Michael Bagnoli, Devon Maranto, Leah Dunn, Duncan McKinney, Eric Steen

**Affiliations:** 1 Surgery, Edward Via College of Osteopathic Medicine, Blacksburg, USA; 2 Internal Medicine, Lewis Gale Medical Center, Salem, USA; 3 General Surgery, Lewis Gale Medical Center, Salem, USA

**Keywords:** cholecystoenteric fistula, robotic-assisted surgery, cholecystectomy complication, bile leak, cholecystoduodenal fistula

## Abstract

Cholelithiasis and its complications are among the most prevalent and costly medical conditions in the United States. Chronic gallbladder disease can progress into more complicated conditions, such as a cholecystoenteric fistula and, more specifically, a cholecystoduodenal fistula (CDF). Repair of these fistulas is complex and usually performed with an open approach. However, if discovered pre-operatively, they can be referred to a hepatobiliary surgery center, where surgeons have specialized training to do such procedures laparoscopically. Here, we present a case of a 57-year-old female with a past medical history of migraines, arthritis, chronic back pain, and fibromyalgia, with no prior surgical history. She presented with an approximately six-month history of colicky right upper quadrant pain and symptomatology consistent with symptomatic cholelithiasis. She elected to have a robotic-assisted laparoscopic cholecystectomy performed. Intraoperatively, she was found to have a CDF and subsequent bile duct leak that were successfully repaired. While more research is required to further characterize and more quickly identify this complication of gallbladder disease, this case highlights the value of robotic-assisted surgery in technically challenging cases. We aim to describe and advocate for the adoption of a robotic approach in patients with comparable presentations, allowing for excellent visualization and control in the removal of inflamed gallbladders, repair of fistulized tissues, and stabilization of bile leaks.

## Introduction

Cholecystoenteric fistulas are a rare yet clinically significant complication of chronic cholecystitis, characterized by a pathologic connection between the gallbladder (GB) and the gastrointestinal tract. The most involved viscus is the duodenum leading to a cholecystoduodenal fistula (CDF) [1]. The formation of a CDF occurs through a sequence of events, initiated by the pressure of a gallstone on the GB wall. This is followed by an inflammatory process of the GB and the development of adhesions with structures surrounding the GB. The stone erodes, via necrosis, through the GB wall into adjacent organs [2-4]. This gives rise to an abnormal connection between the lumen of the GB and the involved structure creating a fistulous tract [5]. High clinical suspicion is necessary for preoperative diagnosis and planning and, given its rarity, it remains difficult despite modern imaging modalities. Patient presentation varies greatly including abdominal pain, diarrhea, weight loss, nausea, jaundice, cholangitis as well as other nonspecific symptoms. The vague presentation of CDFs complicates their identification and the planning of a proper treatment approach [6]. Surgery is the definitive treatment option, but there is no consensus on an optimal approach, although most surgeons opt for an open approach or conversion to an open procedure after a laparoscopic approach is initiated [7]. Due to the inflammation inherent in these patients, extensive adhesions further complicate intraoperative dissection. There are limited reports on the effectiveness of a robotic-assisted laparoscopic approach in this clinical scenario [8]. Despite the advances in recent years, the adaptation of robotic-assisted surgery for the treatment of CDFs remains scarce. Our goal for presenting this case is to further describe the benefits of improved visualization, dexterity, and control the robot offers in hopes surgeons turn to this modality when approaching complex gallbladder surgeries.

## Case presentation

**Figure 1:**
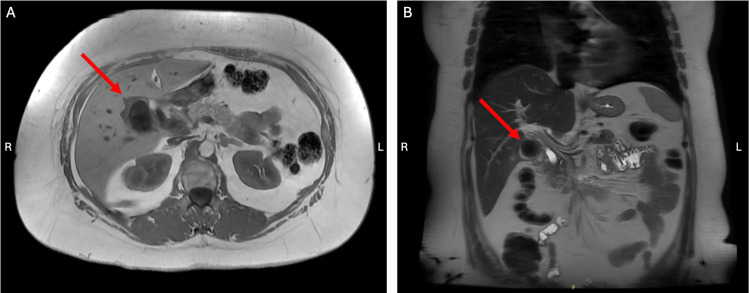
Magnetic resonance cholangiopancreatography (MRCP) Abdominal MRI demonstrating pericholecystic fluid collection and gallbladder wall thickening (red arrows) in the axial (A) and coronal planes (B).

This case details a 57-year-old female with a history of migraines, arthritis, chronic back pain, and fibromyalgia, with no prior surgical history. She presented with an approximately six month history of colicky right upper quadrant pain and symptomatology consistent with symptomatic cholelithiasis. Initial labs were notable for elevated liver enzymes, specifically an alanine aminotransferase (ALT) of 175 U/L and alkaline phosphatase (ALP) of 246 U/L. Magnetic resonance cholangiopancreatography (MRCP) showed a moderately distended GB with a mildly irregular and thickened wall at 4 mm (Figure 1). There were multiple stones within the lumen, the largest stone measuring 3.4 cm in greatest diameter. The common bile duct (CBD) was dilated at 11 to 12 mm in diameter with abrupt distal tapering near the ampulla. The patient was offered a robotic-assisted laparoscopic cholecystectomy to remove her GB for possible symptomatic treatment and was scheduled electively.

A 5 mm vertical incision was made above the umbilicus for initial access. Four robotic ports were placed in the normal fashion for robotic-assisted cholecystectomy and the robot (DaVinci XI, Intuitive Surgical, Sunnyvale, USA) was docked over the patient’s left side. Initially, the GB was obstructed from view by omentum and surrounding visceral adipose tissue. After tedious dissection with the monopolar hook, the dome of the GB was located, and dissection down the GB fossa began. Due to the prominent inflammatory response, intraoperative indocyanine green was not particularly useful in confirming anatomy. The duodenum was found to be overlying and adhering to the anterior wall of the GB thus entertaining the possibility of a fistula. The anterior wall of the GB was dissected to help separate it from the duodenum. After performing an esophagogastroduodenoscopy (EGD), it was clear that a fistula existed between the GB and the duodenum. The fistulized GB and duodenum were divided, the GB excised, and the duodenum was repaired. Before closing, a bile leak was identified and presumed to be coming from the CBD. Upon running the CBD, the leak was indeed identified to be from the CBD and repaired with 4-0 Vicryl. The operative spaces and cavities were irrigated and suctioned for residual bile and blood. Subsequently, a 19 French-Blake drain was placed, and the patient was closed. The total operative time was 5 hours.

**Figure 2:**
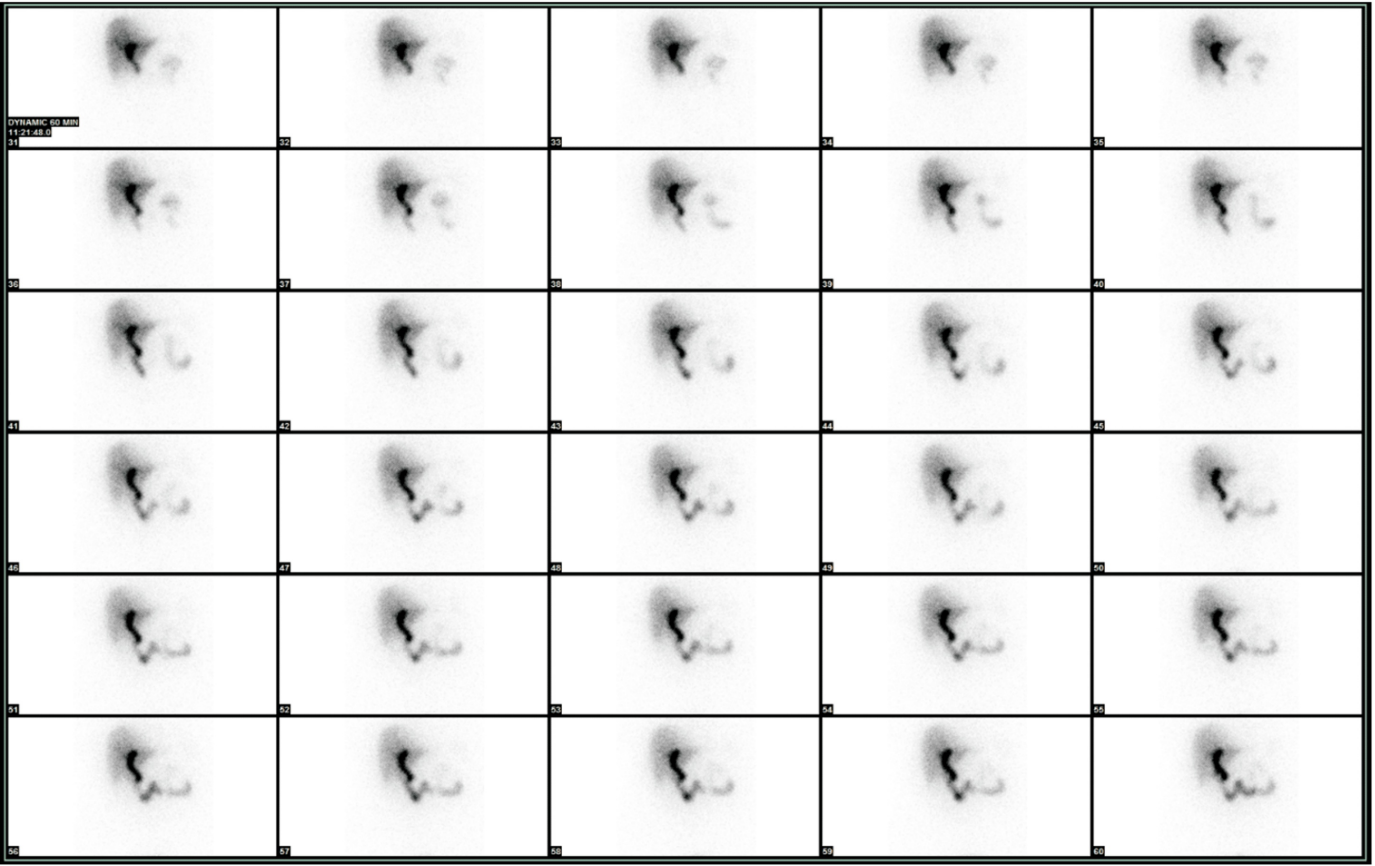
Hepatobiliary iminodiacetic acid (HIDA) scan Cholescintigraphy demonstrating a lack of biliary leakage post-operatively from 31 (top and left-most panel) to 60 (bottom and right-most panel) minutes after radioactive tracer injection.

Postoperatively, the patient was admitted for management with a nasogastric tube and bowel rest. Bile was found in the drain the next day. Octreotide was started and immediately discontinued secondary to an allergic-type reaction experienced by the patient. A 48-hour postoperative hepatobiliary iminodiacetic acid (HIDA) scan revealed no leak, demonstrating the bile leak had ceased after 48 hours without further intervention (Figure 2). The patient was discharged on postoperative day 6 on a normal diet and in stable condition.

## Discussion

Cholecystoenteric fistulas are an uncommon but clinically significant sequelae of gallbladder disease. Described as early as 1890 by Courvoisier, this pathology, which is most commonly secondary to chronic inflammation from cholecystitis, can make the intraoperative environment of cholecystectomy particularly difficult laparoscopically [1,5]. However, in the case presented, we find that the robot allowed us to navigate the inflamed intra-abdominal conditions and unexpected CDF efficiently, safely, and without the need to covert to open. Although we did not attempt laparoscopically, it is unclear whether the laparoscopic approach would have been equally as successful. Regardless, our aim for this case is to encourage the use of robotic-assisted laparoscopy in the setting of potentially complex gallbladder disease. Unfortunately, these complications commonly go undiagnosed preoperatively due to their insidious and potentially asymptomatic nature. Occasionally, these fistulas may be diagnosed via dedicated imaging studies when there is a high clinical suspicion [4].

The most common type of cholecystoenteric fistula is one existing between the GB and the duodenum (71-90%) [5,9]. However, in any case of a cholecystoenteric fistula, there is little consensus on preferred operative management. This is often complicated by the fact that most cases (70-85%) are found intraoperatively, resulting in incomplete surgical planning [1,10]. Whether found preoperatively or during surgery, CDFs have traditionally been managed initially with laparoscopic surgery and then converted to an open approach. It is often included on the consent forms that there is a possibility for any laparoscopic surgery to be converted to an open one. The reason is that certain intraoperative conditions, such as cholecystoenteric fistulas, may pose an increased risk of injury to the patient if the laparoscopic approach is continued. In 2001, a published study suggested that laparoscopic management of cholecystoenteric fistulas was not a contraindication to open surgery [11]. Furthermore, it is described that, in other gallbladder-related laparoscopic surgeries encountering unfavorable intraoperative conditions, converting to an open approach is often the most efficient and beneficial for patient outcomes [12]. In gallbladder disease, success and improved outcomes for the patient population with the laparoscopic approach are well documented. However, it is maintained that converting to an open approach is not a failure in the face of complex intraoperative conditions due to the lower risk of iatrogenic bowel injury, easier suturing, dissection, and shorter overall operative times. [4,13].

The robotic-assisted approach to cholecystectomies has also been shown to be safe and effective [14]. For more intricate hepatobiliary procedures, such as CDFs, it remains uncertain whether a fully robotic-assisted approach is superior or inferior to open or laparoscopic approaches in terms of efficacy and outcomes. There have been few reports on the use of robotic-assisted repair in such entities as CDFs [8]. The first documented robotic repair of a cholecystoenteric fistula was only recently described in 2019 by Baratta et al [4]. Subsequently, from 2022-2023, Krzecowski et al, Hurwitz et al, and Zhu et al describe similar cases of gallbladder disease complicated with cholecystoenteric fistulas in which the robot-assisted approach was undertaken. In these documented cases, it was noted that the robot offered improved dexterity, ergonomics, visualization in the setting of anatomic abnormalities due to inflammation processes, and shorter post-operative stays [15-17]. More recently, in 2024, Alfonso et al assert that with the emergence and publication of cases such as these, the robotic approach may become the recommended method for complex cholecystectomies [8]. 

In congruence with the literature, we describe an additional case that shows the benefits of a robotic-assisted approach. The robot offered exceptional precision, simplifying the differentiation and separation of chronically inflamed tissue between the gallbladder and the intestines. The GB was carefully resected and the fistula was successfully repaired despite numerous, dense adhesions. The additional complication of a bile leak was readily identified and repaired with great precision due to the increased dexterity afforded by the robot. While robotic-assisted surgery may offer superior precision than a laparoscopic approach, it suffers from similar pitfalls such as longer operation times and iatrogenic injuries. With an increasing number of surgeons acquiring proficiency in robotic techniques, complex hepatobiliary procedures, including repair of conditions like CDFs, are becoming integrated into their surgical skill set.

## Conclusions

While the prevalence of cholecystoenteric fistulas is low, their presence is often impactful with significant divergence from preoperative plans, longer intraoperative times, and increased postoperative monitoring. Compounding on their impact is the lack of consensus in the literature regarding the approach to management. We present a case that demonstrates a successful robotic-assisted laparoscopic CDF and bile leak repair without the necessity of converting to an open case. With this case, we aim to enrich the expanding body of surgical robotic literature. The presented case highlights the robot’s ability to effectively handle intraoperative complications, such as CDFs, and subsequently provide valuable insights to surgeons. With robotic-assisted operations increasing in number throughout operating rooms, our goal was to describe an alternative approach for surgeons to have in their armament when surgically managing complex gallbladder disease.
